# Feasibility and Acceptability of a Guided Self-Help, Text-Messaging Intervention to Promote Positive Body Image of Emerging Adult Women

**DOI:** 10.3389/fgwh.2022.849836

**Published:** 2022-04-29

**Authors:** Courtney B. Rogers, Jennifer B. Webb, Lia Bauert, Jordan Carelock

**Affiliations:** ^1^Cherokee Health Systems, Knoxville, TN, United States; ^2^Health Psychology PhD Program, University of North Carolina at Charlotte, Charlotte, NC, United States; ^3^Eating Recovery Center, Denver, CO, United States

**Keywords:** positive body image, mindful self-care, disordered eating, guided self-help (GSH), college women

## Abstract

The predominant approach of existing eating disorder prevention programs targets risk factors for development; furthermore, burgeoning evidence suggests that promotion of protective factors against eating disorders (e.g., positive body image) is also a worthy avenue for prevention efforts. The present study considered existing literature gaps in the design of an 8-week guided self-help intervention meant to address the risk for disordered eating through the improvement of positive body image and enhancement of current adaptive functioning. The intervention incorporated elements of weight-inclusive health promotion (e.g., Health at Every Size; HAES) alongside positive psychology and third-wave behavioral interventions [e.g., self-compassion, mindful eating, Acceptance and Commitment Therapy (ACT)] to promote engagement in mindful-self-care. This mixed-methods study evaluated the feasibility and acceptability of the text-messaging based intervention in a diverse sample of cisgender college women (*N* = 30; 30% Black; 30% bisexual) at risk for disordered eating. Results indicated a high level of engagement and satisfaction with the intervention. Proof of concept was preliminarily supported by the observed significant changes in variables of interest (i.e., body appreciation, positive embodiment, mindful self-care, intuitive eating, self-compassion, disordered eating, and body image dissatisfaction) across the intervention. Overall, results of this study suggest that the use of a guided self-help program based in technology which seeks to reduce risk factors for disordered eating while also supporting adaptive functioning may be indicated for emerging adult women. This article will discuss how the present study provides the groundwork for continued development of innovative and remotely accessible interventions which promote positive body image.

## Introduction

Eating disorders represent a significant burden on the psychological and physiological wellbeing of individuals living in industrialized societies (1). A subclinical presentation of disordered eating (e.g., inappropriate food restriction, binge eating, purging) is associated with a similar amount of functional impairment and distress as its full-blown diagnosable counterpart with an increased prevalence rate (2, 3). Due to the significant negative consequences associated with disordered eating, its treatment and prevention of its development are areas of significant interest (4, 5).

Understanding the risk and protective factors for onset are crucial in designing prevention efforts (6); furthermore, many existing prevention programs focus on addressing risk factors. Regarding course of development, individuals in the “emerging adulthood” period of life [i.e., ages 18–25; (7, 8)] are likely at elevated risk for engagement in disordered eating behaviors (9); further, this is especially true if they identify as female (10). In addition to age and gender, other risk factors for the development of disordered eating include body dissatisfaction, higher weight status, thin ideal and weight bias internalization, and a history of dieting (11). The present study extends our knowledge in the area of prevention to explore how protective factors can be promoted *via* engagement in mindful self-care with the goal of reducing disordered eating behavior and, ultimately, risk for eating disorder development.

Issues of feasibility and acceptability should also be considered in designing interventions with high utility for impacting risk and protective factors (12). Although several prevention programs for eating disorders exist for the emerging adult population and have been demonstrated as efficacious, there are concerns about ongoing availability and the sustainability of these efforts (13, 14). One specific concern with maintaining longevity involves a lack of providers trained to perform these services (15). Moreover, for the emerging adult population, there are also other perceived barriers to treatment to consider such as financial limitations and time constraints (16, 17). For example, although the Body Project has been quite successful in preventing later eating disorder development, several universities in which it was tested discontinued use of the program for reasons such as staff turnover and time limitations for students and staff (14). As a result, there is a need to utilize innovative intervention designs that are both acceptable by this population and have the potential to be efficacious.

One response to the limitations of traditional intervention approaches has been the use of guided self-help intervention (GSH), which involves the provision of materials to individuals that can be applied independently with minimal guidance from a clinician. GSH has been utilized to treat a variety of mental health concerns [e.g., anxiety, depression, stress; (18, 19)] as well as eating disorders (20–23). GSH appears to be uniquely suited to addressing subclinical, disordered eating (24). The delivery style of GSH makes it more accessible than traditional treatment methods and college-aged individuals have rated these types of programs as acceptable [e.g., (21)]. Among existing GSH programs, there has been utilization of virtual technology such as telephone calls [e.g., (25)], online support groups [e.g., (21)], and automated text messaging [e.g., (26)]. To date there has been limited testing of the use of reciprocal text messaging, despite evidence suggesting that young adults prefer this type of communication to other formats [e.g., telephone calls; (27)].

Despite this significant advance in providing accessible, acceptable care, extant GSH programs are limited by factors such as paying primary attention to the reduction of risk factors (rather than promotion of protective factors, such as positive body image and positive embodiment). This continues to be the predominant approach despite support for the inclusion of protective factors as beneficial (28–30). Recent evidence has motivated additional calls for more inclusion of protective factors in ongoing research and practice efforts to impact body image and prevent disordered eating (31). Much of this emerging work is focused on positive body image and embodiment (29, 30, 32–35).

Positive body image is multi-faceted and is conceptualized as consisting of components such as appreciation of the body, responsiveness to the body's needs, and having the ability to effectively navigate threats to body image (35–39). Halliwell (32) identified three potential pathways by which positive body image may protect individuals from negative outcomes: leading to direct improvement in adaptive behaviors (e.g., intuitive eating) and wellbeing, encouraging active avoidance of potentially harmful influences, and enhancing ability to navigate potentially harmful messages and external influences when encountered [e.g., through affect regulation; (40)]. One facet of positive body image which has garnered a significant amount of support for its potential role in this process is body image flexibility.

Body image flexibility represents an adaptive strategy for responding to potentially distressing present-moment experiences of the body. Furthermore, it is an individual's ability to embrace any thoughts, emotions, physical sensations, etc. of their body in an open and non-judgmental manner (41). Body image flexibility can be considered a specialized type of psychological flexibility, which is the primary treatment target of Acceptance and Commitment Therapy [ACT; (42, 43)]. From an affect regulation perspective (40, 44), body image flexibility likely serves as a protective factor due to its value in facilitating flexible and adaptive responding in the face of appearance-related threats and resulting distress. High levels of body image flexibility are associated with decreased eating disorder risk and body dissatisfaction, adaptive eating behaviors (e.g., intuitive eating), body appreciation, and less internalized weight bias [see (45, 46)].

*Embodiment* refers to a sense of ownership over, connection with, and care for one's own body. Furthermore, in embodiment, the body is viewed as a vehicle through which one fully interacts with the world competently and powerfully (33, 36, 47, 48). The enactment of embodying activities is considered crucial for the development of positive body image (36). One way that individuals can participate in these behaviors is through awareness of and responsiveness to the needs of their body (33, 47–49). As a result, researchers and clinicians have been encouraged to focus their efforts on promoting mindful self-care as a means for preventing and treating disordered eating (50). Although mindful self-care is not an aspect of positive body image itself, this practice may provide an avenue through which positive embodiment is supported and development of positive body image can occur (38, 51). Mindful self-care involves a range of behaviors such as engaging in regular physical activity, consuming nutritious foods, eating intuitively, being self-compassionate, and having supportive relationships (28). Aligned with the Attunement Model of Wellness and Embodied Self-Regulation [AMWESR; (50–53)] mindful self-care can support positive body image by serving as a means for balancing and maintaining both internal (thoughts, feelings, and physical body) and external (family, community, and culture) demands and influences on the self. Engagement in mindful self-care requires a shift from focusing on dissatisfaction with the body to fulfilling the body's needs (50) and has been linked to decreased levels of disordered eating behaviors and increased body esteem (28).

### Present Study

This study considered existing limitations in the design of a GSH intervention meant to address factors which increase the risk for disordered eating through the improvement of positive body image and enhancing current adaptive functioning. This mixed-methods study evaluated the feasibility and acceptability of a novel GSH intervention in a sample of college women with pre-existing body image concerns, a group that is at particular risk for disordered eating. This intervention utilized the book *Body Kindness* (54), which encourages improved body image via engagement in mindful self-care behaviors. Participants communicated with a trained support person via text message throughout the intervention. The use of text messaging was chosen to further support acceptability of the intervention due to an increased preference for text message communication by young adults over more traditional GSH formats like telephone calls (27). Prior to this study, this specific body kindness approach had not yet been evaluated. The study had two aims.

#### Aim 1

Test the feasibility and acceptability of an 8-week text message-facilitated guided self-help intervention intended to support participants in making mindful self-care decisions and improving body image. Post-intervention, participants were asked to complete a quantitative feedback questionnaire to measure satisfaction with the intervention. Participants were also asked open-ended questions about what they liked and disliked about the program, recommended changes, and any other feedback they wanted to share. To examine adherence and program engagement, the following were assessed: percentage of attendance at weekly support meetings, attrition from the program, and total number of book activities completed by participants each week.

#### Aim 2

Preliminary efficacy of an 8-week text message-facilitated guided self-help intervention intended to support participants in making mindful self-care decisions and improve body image. This was assessed by examining changes from pre- to post-intervention in body appreciation, body image flexibility, body dissatisfaction, disordered eating, intuitive eating, mindful self-care, positive embodiment, and self-compassion.

## Materials and Methods

### Participants

Prior to participant recruitment, the project was approved by the authors' Institutional Review Board. Participants were 30 women over the age of 18 recruited from a University setting. Participants were deemed eligible if they (a) identified as female; (b) demonstrated significantly elevated body image dissatisfaction and/or low body appreciation; (c) endorsed a desire to improve this experience; and (d) owned a mobile phone or other device that receives text messages. For the purpose of this study, elevated body image dissatisfaction was defined as obtaining a total score ≥ 52 on the Body Shape Questionnaire [indicative of moderate to marked body dissatisfaction; (55)]. Low body appreciation was defined as a score < 2.79 on the Body Appreciation Scale-−2 [one standard deviation below the mean score obtained in a sample of college women; (56)].

Given the low level of care provided by this intervention program, individuals who endorsed the presence of a clinically significant eating disorder or suicidal ideation were excluded. Current eating disorder was indicated by a score ≥ 4 on the Eating Disorder Examination—Questionnaire [given that this placed an individual above the 95th percentile for young adult women; (57)]. Suicidal ideation was indicated by an affirmative response to any of the three questions on the Ask Suicide-Screening Questions [ASQ; (58)]. Those undergoing concurrent treatment to address body image or disordered eating were also excluded. Ineligible participants were provided with information regarding other potential resources (e.g., the University counseling center, National Suicide Prevention Lifeline) as well as the reference for the book used in this intervention.

### Procedure

#### Recruitment

Participants were recruited using email and flyers. Recruitment materials advertised the study as an opportunity to participate in an intervention for women with body image concerns and displayed a link to an online pre-screener. Interested participants completed a screener online to determine whether they met inclusion or exclusion criteria (including a general demographics form and measures of body image and eating disorders). Following within 24 h of screener completion, all prospective participants were contacted *via* email to inform them of whether they met study criteria. Eligible participants were sent an electronic copy of a consent form and were invited *via* email to schedule an orientation session.

#### Trained Support Person

Each eligible participant was assigned a support person, who was either a doctoral student from a clinical psychology program or a graduate of a bachelor's program in psychology. Support persons were trained in the book philosophy and able to aid participants as they completed the intervention. The training was conducted by the lead researcher on this project, a doctoral student, and supervised by a faculty mentor in clinical psychology. Training was based on a modified version of the TeleCoach Manual (59), which incorporates motivational interviewing techniques to elicit participant motivation and commitment to the program, identify and solve potential roadblocks, and improve poor adherence.

#### Orientation and Initial Session

Eligible participants were invited to a location on campus for the orientation session. The purpose of the orientation session was to collect data, inform participants about the intervention, establish rapport with the support person, and elicit motivation to engage in the program. Participants were also provided with a $10 gift card for their time. This meeting lasted ~45 min in total. Following the completion of a hard-copy consent form and a consent quiz to ensure comprehension, participants completed the questionnaire battery to further assess body image flexibility, intuitive eating, mindful self-care, positive embodiment, and self-compassion (given that measures of body appreciation, body dissatisfaction, and disordered eating were already completed in the screening process). All questionnaires were completed on a computer using the Qualtrics software program.

Following the completion of the questionnaire battery, the trained support person described the intervention to the participant and provided the participant with the *Body Kindness* book and a journal. The intervention philosophy was introduced through a discussion of the Introduction and Chapter 1 of the book. Consistent with the Introduction, the support person first described the tenets and rationale of a Body Kindness approach. Chapter 1 of the book focuses on eliciting motivation to engage in body kindness behaviors, initial steps in this process (e.g., not engaging in weighing or calorie tracking), and addressing potential roadblocks to success. The value of journaling reactions to the book was emphasized, and participants were invited to complete their first journaling activity [*Talk Yourself Into Change*; (54)] to explore their motivation to engage with the intervention, the intrinsic benefits of this, and how they would be able to enact change moving forward. Throughout this initial meeting, participants had a chance to ask questions.

#### Intervention

The intervention took place over the course of 8 weeks. Prior to beginning the self-guided intervention, participants completed an in-person visit. During this visit, the participant received materials, met their support person, learned more about the book's philosophy, and had the opportunity to ask any questions. The following 8 weeks were completed primarily independently by the participant, with weekly, virtual check-in meetings with the support person.

##### Body Kindness Book

Participants were assigned the self-help book *Body Kindness* (54) to read. The book is separated into three major sections: what you do, how you feel, and who you are. It strongly incorporates elements of positive psychology and third-wave behavioral interventions such as acceptance, mindfulness, psychological flexibility, values-based behavior, and encouragement of self-compassion. It is written from a body-positive stance and encourages a more weight-inclusive approach to wellness [as compared to a weight-loss paradigm: (60)].

##### Journaling

Each section of Body Kindness includes a variety of journaling prompts and activities to complete. To assist participants in completing these activities, they were provided with a journal to utilize each week. Given that this study aimed to explore the initial feasibility and acceptability of the book, participants were asked to only complete a minimum of one activity from the book per week; however, they were asked to indicate the activities they attempted each week during their weekly support exchange. Participants were given the option to share a de-identified copy of their journal pages at the end of the intervention. To encourage authentic responding in the journal entries, refusal to share the journal pages did not exclude participants from taking part in the study.

##### Support Meetings

The assigned support person contacted the participant weekly via text message. Contact occurred at two different time points throughout each week: first, a standardized reminder or message of encouragement was sent to participants mid-week. At a second time point during the week, the pre-scheduled text exchange occurred, lasting a maximum of 30 min. The purpose of each session was to check in with the participant about the previous week, identify any barriers and troubleshoot concerns, and to prepare participants for the upcoming week of the intervention. The support person followed a semi-structured script to ensure that all necessary elements were covered, as well as to maintain a degree of standardization. Participants were informed that the text account was not monitored for messages outside of the scheduled session time. Supporter fidelity was checked by the lead researcher through a review of a random support session transcript in weeks 1, 3, 5, and 8. Overall, fidelity was high with 87.5% of sessions following the supporter guide.

#### Follow-Up

Following the completion of the intervention, participants were asked to complete another in-person visit to complete follow-up questionnaires. Questionnaires were again administered on computers to assess body appreciation, body dissatisfaction, eating behaviors, mindful self-care, self-compassion, and positive embodiment. Feedback from participants about the program (both qualitative and quantitative) was also collected. Participants were provided a $15 gift card at this meeting as compensation for their time.

### Measures

Data collection for the study occurred from September to December 2019 prior to the onset of the COVID-19 pandemic. An additional, secondary measure of weight bias internalization was also collected at this time.

#### Demographics

The following demographic variables were assessed: age, gender, race/ethnicity, sexual orientation, academic rank and major, height, weight, socioeconomic status (*via* mother's educational attainment), current employment status, history of psychological treatment for body image and eating behaviors, dis/ability status, and extent of prior experience with self-help approaches.

##### Suicidal Ideation

As part of screening procedures, potential participants were assessed for suicidal ideation using three questions from the Ask Suicide-Screening Questions [ASQ; (58)]. Questions included “In the past few weeks, have you wished you were dead?” “In the past few weeks, have you felt that you or your family would be better off if you were dead?” and “In the past week, have you been having thoughts about killing yourself?” These questions could be answered with either Yes or No responses. An affirmative response to any of these three questions was considered sufficient to render the individual ineligible for participation and led to the provision of further resources.

#### Body Appreciation

Body appreciation is defined as favorable evaluation of the body, despite actual physical appearance (61). Body appreciation was measured using the Body Appreciation Scale, second edition [BAS-2; (56)]. The scale consists of 10 items which are answered using a 5-point Likert scale (1 = *never*, 5 = *always*) to indicate how frequently they engage in a given action. For example, as part of this questionnaire, participants will respond to questions such as, “I appreciate the different and unique characteristics of my body.” The items are summed with higher scores indicating greater levels of body appreciation. Among a large sample of men and women from a college setting and an online community, the BAS-2 was found to be internally consistent (α = 0.86 in the present study), test-retest reliable, and to have construct validity.

#### Body Dissatisfaction

Body dissatisfaction is an evaluative component of body image (62, 63) and has been noted as a crucial risk factor for engagement in maladaptive eating behaviors and eating disorder development (29, 64–67). The shortened, 16-item version of the Body Shape Questionnaire [BSQ; (55, 68)] was used to assess satisfaction with one's body. The BSQ asks participants to respond to a series of questions asking how often they have had negative experiences of their body over the past 4 weeks using a 6-point Likert scale (1 = *never*, 6 = *always*). An overall score is obtained by adding up scores on the items. Higher scores indicate greater body dissatisfaction. The BSQ has been demonstrated as internally consistent (α = 0.92 in the present study), test-retest reliable, and as having construct validity in a sample of college women.

#### Body Image Flexibility

The Body Image Acceptance and Action Questionnaire [BI-AAQ; (41)] was utilized to measure body image flexibility. The BI-AAQ consists of 12 items (e.g., “Worrying about my weight makes it difficult for me to live a life that I value”) which are responded to using a 7-item Likert scale (1 = *never true*, 7 = *always true*). Responses are summed, with higher scores indicating increased levels of body image inflexibility. The BI-AAQ has been demonstrated to be internally consistent and reliable over a 2 to 3-week time period (α = 0.90 in the present study).

#### Disordered Eating

The 28-item Eating Disorder Examination-Questionnaire [EDE-Q; (69, 70)] was utilized to screen for potential disordered eating behaviors and monitor changes in eating behaviors over time. The measure includes four subscales (restraint, eating concern, shape concern, and weight concern) which can be averaged to a score for each. Further, the four subscales can also be averaged to obtain a global score. Higher scores indicate increased disordered eating symptomatology. Participants responded to questions such as, “Have you tried to follow definite rules regarding your eating in order to influence your shape or weight?” to indicate the frequency of a given behavior over the past 28 days using a variety of response styles (e.g., Likert type, write-in). The EDE-Q subscales have been demonstrated to be internally consistent and test-retest reliable in a sample of college women [(71); α = 0.86 in the present study for the global score].

#### Intuitive Eating

Eating in a manner which is attuned with the body's hunger and satiety cues was measured using the Intuitive Eating Scale-−2 [IES-2; (72)]. The measure asks participants to indicate their level of agreement with 23 items assessing their attitudes and behaviors related to eating (e.g., “I find myself eating when I am lonely, even when I'm not physically hungry”) using a 5-point Likert Scale (1 = strongly disagree, 5 = strongly agree). The IES-2 consists of four subscales (unconditional permission to eat, eating for physical rather than emotional reasons, reliance on internal hunger/satiety cues; body-food choice congruence), and a total score can also be obtained by summing the items and dividing by 23 (after reverse-scoring seven items). The scale is demonstrated to have acceptable psychometric properties in college students [(72); α = 0.82 in the present study].

#### Mindful Self-Care

The Mindful Self-Care Scale [MSCS; (28)] was utilized to measure engagement in self-care behaviors. As written, the scale asks participants to respond to 33 items with their frequency of a given behavior over the past week using a 5-point Likert scale (1 = *never*, 5 = *regularly*). To account for intervention length, the scale instructions were modified to ask about engagement in these behaviors over the past month. The MSCS measures six factors: physical care (e.g., through healthy eating, physical activity), supportive relationships, mindful awareness, self-compassion and purpose, mindful relaxation, and supportive structure. After reverse-scoring one item, the scores in each subscale are averaged. To obtain a total score, the subscale total scores are averaged again. The scale was validated in an age-diverse sample of women, has high levels of internal consistency at both the scale and subscale levels (total scale α = 0.90 in the present study), and correlates with other constructs as expected [i.e., inversely correlated with disordered eating and positively correlated with body esteem; (28)].

#### Positive Embodiment

Embodiment was measured using the Physical Body Experiences Questionnaire [PBE; (73)]. The PBE includes 18 items that assess the extent of participant physical embodiment (e.g., “I respect my body's physical limits”) using a 7-point Likert scale. The scale consists of four subscales (Mind/Body Connection, Body Acceptance, Physical Competence, and Physical Limits), but a total score can also be obtained. Following reverse-scoring of two items (1 and 16), items are averaged with higher scores indicating a greater level of embodiment. The PBE has been demonstrated to maintain acceptable psychometric properties in a sample of undergraduate women (α = 0.86 in the present study).

#### Self-Compassion

The Self-Compassion Scale—Short Form [SCS-SF; (74)] is a 12-item questionnaire that measures an individual's level of self-compassion. The scale consists of six subscales which represent the multidimensional construct of self-compassion (common humanity, isolation, mindfulness, over-identified, self-kindness, and self-judgment); however, a total self-compassion score is also provided. Participants were asked the frequency with which they engage in a given behavior and rank their answer on a 5-point Likert-type scale (1 = almost never; 5 = almost always). Responses to these items were summed, with lower scores indicating a reduced level of self-compassion. The total score for SCS-SF has been demonstrated as having similarly strong psychometric properties as compared to the full-scale version [although the subscale scores have lower reliability; (75); α = 0.77 in the present study].

#### Feasibility and Acceptability

Participants were also asked to complete a series of Likert-scale and open-ended questions to assess feasibility and acceptability of the program. This questionnaire incorporated questions from the Feasibility and Acceptability of Intervention Measures [AIM; FIM; (76)] as well as questions used in focus groups examining feasibility of the Body Kindness book for postpartum women (Webb et al., n.d.). The AIM and FIM have been demonstrated to have strong psychometric properties; however, the predictive validity of the measures is still under evaluation. Sample questions from the AIM and FIM include “The program seems doable” and “The program meets my approval.” Additional open-ended questions assessed perceived strengths and weaknesses of the program, whether participants would recommend this to another person, and what participants would change about the program.

To further examine adherence and program engagement, the percentage of support sessions attended was calculated (including the percentage of attrition from the program). The mean number of activities completed by participants each week (as indicated in the support exchanges) and the percentage of participants completing at least one activity per week were also assessed.

### Analysis

This study utilized a single-group pre-post design. Both quantitative and qualitative analyses were utilized to investigate feasibility and acceptability of the intervention program.

#### Aim 1 Analyses

Feasibility and acceptability were assessed both quantitatively and qualitatively. Quantitative analyses were conducted using the Statistical Package for the Social Sciences (IBM SPSS v. 25) to examine responses to the participant feedback questionnaire. Descriptive statistics were computed and analyzed for each question (as well as the total FIM and AIM scores) to ascertain participant reactions to the intervention.

Qualitative analysis was also used to examine the open-ended feedback questions to better understand the feasibility of the intervention program. A Hypothesis Coding approach was used to identify patterns in participant responses to the program (77). This process involved the development of a codebook in advance of data collection. The codes were based on expectations about the data that would be collected (e.g., codes such as likes, dislikes, recommended changes). Following data collection, participant responses were coded by the lead researcher. Codes were then analyzed using frequency counts.

#### Aim 2 Analyses

Quantitative analyses were conducted using the Statistical Package for the Social Sciences (IBM SPSS v. 25) to examine preliminary participant response to the intervention (i.e., proof of concept). Descriptive statistics were computed for all variables. Given the preliminary stage of analysis, no outcome measure was set as primary. Paired *t*-test analyses, along with reported effect sizes in the form of Cohen's *d*, were used to examine changes from pre- to post-intervention in each construct of interest (i.e., body appreciation, body dissatisfaction, disordered eating, intuitive eating, mindful self-care, self-compassion, and positive embodiment).

## Results

One hundred and sixty-nine women expressed interest in participating in the study by emailing the address in the study advertisements. Forty-seven of these individuals did not complete the screener survey sent to them, and 74 were deemed ineligible (*n* = 17 did not endorse elevated body image concerns; *n* = 30 endorsed clinically significant eating disorder symptoms; *n* = 8 were enrolled in concurrent treatment; *n* = 40 responded affirmatively to the ASQ). Forty-eight women who met criteria for the study were invited to participate, and 30 participants formally began the program. Of those that were invited to participate but did not enter the program, 16 did not respond to the invitation to schedule their orientation. Two did not attend the orientation after scheduling and, as a result, others were invited to attend in their place. Participants that did not formally begin the program were provided with information about other mental health resources.

### Demographics

Among the 30 participants that began the program, the average age was 20.1 years (*SD* = 1.67; age ranged from 18 to 24). A majority of these participants identified as either White (40%) or Black (30%). The average BMI was 28.54 (*SD* = 5.36). Although a majority (70%) of participants identified as heterosexual, 30% (*n* = 7) indicated that they identified as bisexual. Other participant characteristics can be seen in Table 1.

**Table 1:** Participant demographics.

		***M/%***	***SD/n***
Age		20.10	1.67
BMI		28.54	5.36
Race/ethnicity	White	40	12
	Black	30	9
	Asian/Asian-American	13.3	4
	Multiracial	10	3
	Hispanic/Latina	6.7	2
Sexual orientation	Heterosexual	70	21
	Bisexual	30	9
Relationship status	Single	70	21
	In a relationship	26.7	8
	Divorced	3.3	1
Dis/ability status	None reported	73.3	22
	Mental disability	16.7	5
	Physical disability	3.3	1
Academic rank	Freshman	16.7	5
	Sophomore	40	12
	Junior	23.3	7
	Senior	16.7	5
	Graduate student	3.3	1
Mother's educational attainment	Incomplete high school	10	3
	High school/GED	16.7	5
	Some college	40	12
	Bachelors	26.7	8
	Graduate Work	6.7	2
Employment status	Student not employed	40	12
	Student working part-time	60	18
Prior experience with self-help	Experienced	6.7	2
	Somewhat experienced	33.3	10
	Inexperienced/no experience	60.0	18

*N = 30*.

### Aim 1: Feasibility and Acceptability

#### Study Attrition

Participants were considered withdrawn from the study if they notified the study coordinators that they were no longer interested or missed three consecutive support sessions. One participant withdrew at each of weeks 4, 5, and 6 and two participants withdrew at week 7. Three of these withdrawals were due to lack of engagement with the study (i.e., participants did not respond to 3 weeks' attempts of contact), one was due to a family emergency, and one was due to an expressed lack of time. In total, 25 (83.3%) participants completed the program through Session Eight, and 22 (73.3%) attended the follow-up session. One participant failed to complete the post-treatment questionnaires at the follow-up session; as a result, they were excluded from the dataset, and the final per-protocol analyses included a total of 21 completers.

#### Study Engagement

Participants consistently attended text sessions, with 90.4% of sessions attended throughout the study. On average, these sessions lasted 28.1 min. Participant engagement in book activities was assessed by supporters at each text session. Participants were asked to complete at least one activity per week, and the mean number of activities completed each week was 1.40 (mode was 1 in all weeks except week 4 when the mode was 2). Across the entire program, participants reported completion of at least one activity per week in 84.2% of attended sessions. The range of activities completed varied by week; for example, in week 4, one participant completed 6 activities which was the highest number of any week. It is relevant to note that, in some weeks, there was only one activity available to complete.

#### Feedback Questionnaire

Both quantitative and qualitative analyses were utilized to examine participant attitudes toward the intervention program. Quantitative analysis was utilized to understand participant response to the Likert-style questions on the questionnaire. Descriptive statistics were computed and analyzed for each feedback question (as well as total scores for the FIM and AIM scales) and can be seen below in Table 2. Overall, participants endorsed favorable views of both the book and the program as a whole.

**Table 2:** Feedback questionnaire data.

**Statement**	***M***	***SD***
I found the book readable.	4.85	0.37
I found the writing style in the book engaging.	4.70	0.47
I found the book visually appealing.	4.85	0.37
I found the examples used in the book relatable.	4.55	0.61
I would prefer an electronic copy of the book to the hard copy.	1.60	0.82
I would recommend the book to others.	4.55	0.51
This program improved my body image.	3.85	0.81
This program helped me to be kinder to myself.	4.30	0.66
I believe that this program would be helpful for other women my age.	4.60	0.50
I believe that this program is doable given the constraints in my life.	4.50	0.51
I had enough time to read the assigned book chapters each week.	4.45	0.76
I had enough time to complete the book activities each week.	4.45	0.76
The weekly communication with my support person was helpful.	4.45	0.69
I received the right amount of messages from my support person.	4.50	0.51
I would recommend this program to others.	4.60	0.60
AIM	4.61	0.43
FIM	4.63	0.46

*n = 21*.

Qualitative analysis was used to examine the open-ended feedback questions. Consistent with the hypothesis coding approach (77), codes were created prior to data collection based upon expected responses. Feedback fell into seven different categories: likes, dislikes, perceived barriers to participating in the program, suitability for college women, perceived benefits of the program, recommended changes, and why the participant would (or would not) recommend the program to others.

##### Participant Feedback: Likes

When asked about what they liked about the program, a number of participants discussed their support person. Thirteen participants indicated that they enjoyed having the text sessions with their support person throughout the program. For instance, one participant wrote, “I really enjoyed the constant communication between my support person and me. It truly was motivating and created the enthusiasm needed to continue with the reading.” Five participants noted that the interaction with their supporter enhanced their engagement with the program and made them feel more motivated/accountable; for example, one person wrote, “I believe the support person was a phenomenal idea and can create long-lasting feelings of encouragement.” Five participants stated that their support person assisted them with useful information or advice throughout the program.

Several participants also commented on the book itself. Six participants reported that the book provided useful information in a way that they could apply it to their lives. For example, one participant wrote, “I liked the book itself the most about the program because I think it has some really positive messages that a lot of women need to hear.” Another said, “I loved reading the chapters and then going back and doing the spiral-ups because they helped me better relate the information to my life.” Two participants indicated that they enjoyed the flexible nature of the book. The writing style was noted as enjoyable by two participants, and one person commented on the visual appeal.

##### Participant Feedback: Dislikes

When asked about dislikes, 10 participants reported that there was nothing they disliked about the program and eight participants left the question blank. Three participants noted that, in certain weeks, it felt as if there was “a lot” of reading and they did not have the time to complete it. Three participants commented on the support sessions for various reasons; for instance, one participant stated that they would have liked to be able to reach out to their supporter between sessions if needed. Another participant wrote, “…some of the weeks the checkups felt more script-like than others, which I know is probably because there are certain questions that must be asked.” A third participant noted that they did not like having to speak to a different support person in a week they rescheduled from their normal support time.

##### Participant Feedback: Perceived Barriers

When asked about perceived barriers to participation in the program, the most frequently cited concern was time management. Thirteen participants commented on this issue, and multiple individuals noted it was difficult to balance engagement with their schoolwork. For example, one wrote, “I think it was more of a time-management issue, in which it was difficult sometimes to do this program as well as study for exams and do assignments.” Two participants noted that they had a difficult time finding the motivation to engage with the book. One of these participants reported, “…wanting to self-sabotage instead of practicing body kindness.”

##### Participant Feedback: Suitability for College Women

Participants were asked how well they believed this program meets the self-care needs of college-aged women. Overall, participants appeared to have a positive opinion of this aspect of the program, with 16 participants indicating a belief that it sufficiently addresses the needs of this group. Several of these individuals noted that the guidance of the program felt “manageable” or “doable.” Regarding both the book and the program as a whole, seven participants noted that it felt personalized and/or relevant to their lives. One participant wrote, “I think the program was spot-on with the self-care aspects because most of the concepts discussed are issues that can be seen every day.”

On the other hand, a few participants also commented on the lack of relevance of the book material to their lives. Two participants described this as a barrier to their engagement. One wrote, “I feel like some topics were about things I haven't experienced yet or maybe just see in a different way than a more established woman would.” Three participants noted that this was what they disliked most about the program; for example, one participant wrote, “Some of the examples didn't always help me and the author seemed to make some assumptions about having friends and a support system.” This concern about relevance also was raised when asked how well this program meets the needs of college-aged women. Three responses to this question noted that the book content felt more suited to a mature audience. For instance, one person stated, “I could tell [the book] was clearly geared toward older, middle aged women.” Although participants did not elaborate on why they believed this was the case, it may be due in part to occasional book references to a partner or children.

##### Participant Feedback: Perceived Benefits

When asked to identify any perceived benefits of the program, a notable number of participants pointed to the skills they had learned. Seven individuals commented on the benefit of learning strategies to utilize in the future; for example, one participant wrote, “I am now able to go to something to practice body kindness when my body image is poor. I have practical choices for body kindness to choose from.” Participants also shared how the program helped them to learn strategies not directly related to body image. One participant believed that they had improved their time management skills, and another shared that they had been able to save money by using strategies in the book.

Participants also reflected on how the program impacted their outlook on self-care and body image. Ten participants shared that the program had benefited them by increasing their feeling of love, appreciation, and/or positivity toward themselves. One of these participants wrote, “Honestly throughout this program I was better able to think about my self-care and how I need to appreciate myself more often. It was a refreshing viewpoint and brought back a positive and happier me.” Two participants expressed that the program had taught or reminded them that they were not isolated in experiencing body image concerns; for instance, one stated, “I think I was reminded many times throughout the semester that everyone is trying the best they can, including me, and this was helpful to hear from someone else's perspective.” Five participants also commented, more generally, on how the program had changed their perspectives on a variety of topics.

##### Participant Feedback: Recommended Changes

Overall, many participants appeared to be satisfied with the current state of the program: seven participants wrote that they would not change it, and two left the question blank. Of those that did make suggestions, three participants commented on the support person. Two of these individuals recommended that there be more flexibility in meeting time and type (i.e., ability to meet in person or communicate outside of predetermined times if needed). One individual suggested that the support person could be an optional feature. Other participants commented on the format of the program; for instance, one shared, “My only suggestion would be to make it longer” while another participant wrote that an online diary would be a desirable change. Two individuals suggested that they would like to meet other participants in the program for support. One participant noted that they would recommend improving the relevance of the book content for a wider variety of diets (e.g., vegetarian diet). Lastly, one participant wrote that they would prefer more guidance on which book activities to complete each week, and another stated that they would like there to be less reading.

##### Participant Feedback: Recommending to Others

All participants that completed the questionnaire responded that they would recommend this program to others, and two participants explicitly stated that they had already shared about the program or the book with others in their lives. When asked to provide their reasoning for recommendation, 19 wrote that they believed the book was beneficial to them and/or would help others. Three of these individuals shared their belief that anyone could benefit from the knowledge in the program. Three others wrote that they would recommend the program because it was enjoyable to complete. A few participants also commented on the type of person that they believed would benefit most from the program. Three noted that the program is especially useful if you have the time to devote to completing it. Two others shared that they believed a wider variety of individuals, such as men or adolescents, would also benefit from participation.

### Aim 2: Preliminary Participant Response to Intervention

To further examine participant response to intervention, changes from pre- to post-intervention in each construct of interest (i.e., body appreciation, body dissatisfaction, disordered eating, intuitive eating, mindful self-care, self-compassion, and positive embodiment) were examined after use of SPSS to perform five multiple imputations to handle missing data among program non-completers. Means, standard deviations, *t*-scores and effect sizes (in the form of Cohen's *d*) for each of these can be seen in Table 3. Following the 8-week GSH intervention, significant changes (*p* < 0.01) were seen for each measured outcome, with large effect sizes observed (*d* ranging from 1.26 to 2.67).

**Table 3:** Pre- and post-intervention means, standard deviations, *T*-scores, and effect sizes for variables of interest.

	***M (SD)***	***T***	***D***
Body appreciation	Pre: 23.73 (5.64) Post: 36.40 (3.97)	−7.44^**^	−1.36
Body dissatisfaction	Pre: 67.30 (7.04) Post: 36.67 (10.75)	14.63^**^	2.67
Disordered eating	Pre: 67.07 (15.28) Post: 35.38 (17.48)	8.65^**^	1.58
Intuitive eating	Pre: 2.79 (0.34) Post: 3.44 (0.40)	−8.88^**^	−1.62
Mindful self-care	Pre: 2.78 (0.56) Post: 3.48 (0.28)	−6.89^**^	−1.26
Positive embodiment	Pre: 4.20 (0.70) Post: 5.15 (0.72)	−7.11^**^	−1.30
Self-compassion	Pre: 2.42 (0.51) Post: 3.38 (0.41)	−8.27^**^	−1.51

*N = 30*.

** *p < 0.01*.

## Discussion

To account for limitations of past prevention programs, the present study explored the feasibility and acceptability of a novel, 8-week GSH intervention intended to impact both risk and protective factors for eating disorder development in diverse emerging adult women. Grounded in Cook-Cottone et al.s' AMWESR model (50–53), this program utilized a self-help book incorporating elements of Acceptance and Commitment Therapy (ACT), self-compassion, mindful eating, and positive psychology to support engagement in mindful self-care behaviors. Each week, participants were asked to read an assigned section of the book, complete one activity of their choice, and communicate with a trained support person. The novelty of this program was further enhanced using text messaging for communication, which is arguably a more acceptable modality for the emerging adult population as compared to phone calls (27, 78).

Overall, the present study findings indicate that the use of a GSH program, which seeks to reduce risk factors for eating disorders while also supporting adaptive functioning through a mindful self-care approach in a scalable and accessible way, may be indicated for emerging adult women. Further discussion of the findings and implications for future research, clinical practice, and theory are presented below.

### Aim 1: Feasibility and Acceptability

The first aim of this study was testing the feasibility and acceptability of the GSH intervention. Across both quantitative and qualitative feedback data, participants endorsed favorable views of the program. Altogether, the data suggest that this intervention is both feasible and acceptable for the emerging adult population.

#### Participant Opinion

Participants endorsed positive attitudes of the program across both quantitative and qualitative measures. In particular, participants indicated that they enjoyed working with their support person and found this component of the program motivational. This is consistent with what has been reported in past GSH program investigations for various psychological concerns (79). One concern with traditional programs is the lack of trained professionals to implement these approaches (23). Supporters in the present study possessed a range of background knowledge in psychology but were each trained in accordance with a modified coaching guide (which was followed with a high level of fidelity). Results provide initial support for the use of trained paraprofessionals in this type of approach, which may lend to program sustainability when implemented in real-world settings.

Although most participants endorsed finding the book content enjoyable and relatable, one concern noted by a few individuals was that at times the program felt designed for a more mature audience. Emerging adulthood is a period marked by unique life concerns, such as burgeoning independence and establishment of identity (7, 8). Furthermore, although much of the book content can likely be generalized to a wide audience, suggestions based on having long-term relationships, stable access to resources such as kitchen facilities, or assuming financial security may preclude some individuals from identifying with the book at this point of the lifespan.

#### Program Adherence and Engagement

Overall, participants displayed a high level of engagement with and adherence to the intervention program. This was demonstrated by consistent attendance at virtual support sessions (i.e., 90.4% of sessions attended) as well as a high level of reported completion of at least one activity per week. Nearly three-quarters of participants completed the entire program (including the follow-up session), which is consistent with past evidence suggesting that programs with coach assistance often demonstrate strong participant retention (80). The high level of completion seen in the current study lends further evidence to the feasibility of this approach.

In past investigations, time management has been a noteworthy barrier to accessibility to and engagement with prevention programs for emerging adults (16). Unsurprisingly, this was also a prominent consideration in the present study as well. In the quantitative portion of the feedback questionnaire, participants who completed the study indicated on average that they believed that this program was doable given other time constraints in their life; however, many participants also pointed to time management as a barrier to their participation in qualitative feedback. Despite these concerns about time management, there was still a considerable level of engagement with the program, which suggests that perhaps small modifications (discussed further in the Future Implications section) could improve participant perceptions of this aspect.

### Aim 2: Initial Response to Intervention

The second aim of this study was exploration of the preliminary efficacy of the program. This was examined by assessing changes from pre- to post-intervention in measures of the constructs of interest. Across each construct, significant changes were observed (with large effect sizes) in their respective self-report indicator. Although the mindful self-care approach has been recommended in the past as a valuable adjunct to disordered eating treatment (50), this is the first time in which it has been explicitly utilized in a GSH format. These results provide further indication that the use of a mindful self-care approach can serve to not only support adaptive functioning but also to mitigate risk factors for disordered eating.

Unsurprisingly, given the focus on encouraging mindful self-care behaviors by the utilized self-help book, participants endorsed a significant increase in these behaviors across the study. This was accompanied by a similar reported improvement in the experience of positive embodiment, which could be conceptualized as being enacted through mindful self-care behaviors. Consistent with theory about engagement in mindful self-care supporting positive body image, significant improvement in body appreciation was also observed across the intervention. Interestingly, participants also described their perceptions that these positive changes had occurred in the feedback data. Although the prior study of GSH programs explicitly utilizing a mindful self-care approach is limited, the results of this study point to the feasibility of delivering this approach in a self-guided format.

The positive changes displayed by participants in this study may also be consistent with observations in other GSH studies utilizing an ACT-based approach to enhance body image and adaptive functioning [e.g., (81)]. Participants in the present study were exposed to elements of both ACT and mindful self-care, which are largely compatible in theory and approach. Like the mindful self-care approach, ACT encourages a focus shift toward expressing values through intentional behavioral acts (43). Both also support a focus on functionality. Results of a 2019 study examining the impact an online writing task requiring participants to consider positive aspects of their appearance and body functionality showed that encouraging this shift in focus is also associated with improvements in positive body image (82).

Improvements demonstrated by participants in the present study can also be considered in relation to those seen in other GSH programs focused on self-compassion training. Similar to studies by Toole and Craighead (83), Donovan et al. (84), Sommers-Spijkerman et al. (85), and Rodgers et al. (86), participants in this study experienced improvements in both positive body image and self-compassion. Although the Body Kindness book is not solely comprised of compassion-focused material, it does include a chapter solely dedicated to the concept. An emphasis on self-compassion is also observable throughout other chapters. This may suggest further testing is warranted of the Body Kindness approach to also impact self-compassion.

In addition to improvements in adaptive functioning, participants in the present study also demonstrated changes in maladaptive behaviors and processes across the intervention. Most notably, large changes were observed in body image dissatisfaction; further, improvements were also observed in disordered eating. Results of this study further support the idea that encouraging engagement in adaptive coping can mitigate more negative processes. It is possible that promoting a focus on tending to the self through mindful self-care necessitated participants to shift away from dissatisfaction with their bodies and subsequent maladaptive strategies to change it [e.g., through dieting; (50)].

### Strengths and Limitations

The present study had several notable strengths. First, the sample was diverse in race/ethnicity, sexual orientation, and socioeconomic status as compared to other prevention studies [e.g., see (87) for more information on the lack of racial/ethnic representation in prevention studies]. Another strength of this study was the balance between novelty and evidence base in the approach utilized. Although the book incorporated elements of evidence-based treatments such as ACT, this was the first study of its kind to incorporate an expanded focus on mindful self-care behaviors in the context of a GSH approach.

The design of the study is also noteworthy as a strength (outside of the typical constraints of this type of feasibility study, explored below). To measure constructs of interest, measures were utilized which have strong psychometric properties in an emerging adult population. Moreover, research assistants were well-trained using established coaching guides and fidelity checks were incorporated to ensure no additional training was necessary throughout the study. Lastly, multiple methods were utilized for data collection. This allowed for a richer picture to be painted regarding participant opinion of and response to the intervention program and directly impacts future recommendations for implementation through use of more descriptive participant feedback.

It is also important to acknowledge the limitations of the current study and how these may impact interpretation and generalizability of results. First, there are inherent limitations to the single group, pre-post design. Namely, this includes lack of randomization, which should temper interpretation of results (especially regarding changes over time in constructs of interest). Lack of control in some areas (e.g., in some weeks where participants needed to reschedule, a different supporter filled in) is also notable. Even without a control group, running several groups (each beginning at a different time point) might have been a better test of how feasible this intervention is given that participant engagement may vary as a result of factors such as time of the semester.

Another significant limitation of the current study was lack of participant follow-up. Because this was not incorporated into the study design, it is unclear whether improvements seen by participants were sustained over time. There were also no attempts to contact participants to learn why they may have withdrawn from the study. Although one participant did share that this was due to family concerns, it is unclear why others may have withdrawn at other various time points. This lack of follow-up should further temper the interpretation of the results of the study, as it is possible that certain barriers were not explored from those participants that did not complete the intervention. Future studies would benefit from follow-up for participants that dropped out as well as those that completed the study to understand long-term impacts.

Participant dropout, especially following week 8 prior to the follow-up session, also created a concern about missing data. It is likely that this dropout occurred, in part, as a result of the coinciding timing of the follow-up sessions with the University's final exam period; however, it is still unknown whether these additional data would have painted a different picture with regard to the feasibility and acceptability of this approach. Future studies may wish to consider an assessment process which is entirely online in an effort to improve retention of participants and minimize the gap between the end of treatment and measurement of outcomes.

### Future Directions and Implications

#### Implications for Research

This study provides the groundwork for future larger-scale intervention investigations based in the current approach. Models for successful intervention development call for programs to be scaled over time in order to create approaches which are both efficacious and maximize external validity (12). Based on the results of this study, next steps for intervention development may involve more traditional testing of intervention efficacy, followed by implementing this approach in a rigorous manner within existing systems (e.g., offered through college counseling centers). In addition to incorporating more rigorous control, dismantling studies examining active ingredients and mechanisms of change may also add value in this line of research.

Based on the results of this study, future iterations of this intervention program may benefit from certain changes. For instance, regarding format of the program, changes that improve the flexibility of implementation may be desirable to individuals. Providing the option of asynchronous text messaging (vs. fixed timing of sessions) is one potential avenue to consider. Changing the length of the program, as well as the amount of content provided to participants, could also be tested to lighten burdens on participants' time. Other changes may be made to improve the utility of the program, such as putting some elements online (e.g., the journal). This would also provide a rich source of data for researchers to examine. Related to improvement of data, more frequent measurement throughout the study (e.g., a mid-treatment assessment, delayed measurement following final sessions to examine long-term nature of effects) is also a desirable modification.

The present intervention could also be modified to further enhance elements which many participants already reported enjoying. Given that participants found the supportive elements of coaches to be a strength, incorporation of a support group among participants may expand upon this aspect. The relevancy of the content is another aspect to consider. Although most participants found the book relatable, adapting the principles in the book to more directly address common concerns of emerging adults attending college would likely be a welcome change. Moreover, providing a disclaimer in the orientation session that not all content may feel relevant to each participant could be another strategy for addressing this participant perception.

Given that a high number of individuals expressed interest in participating in the present study but were ineligible for a variety of reasons, future related studies may also want to consider if the eligibility criteria could be expanded in a safe and appropriate manner. The major reasons for ineligibility included suicidal ideation, clinically significant eating disorder symptoms, and lack of body image distress. For example, it is possible (especially for those already receiving treatment) that a GSH approach such as the one used in the present study could be tested as a supplement to their existing treatment plan. This would provide a safety net in case the approach used in the present study is not adequate to address a higher level of care.

Lastly, although the use of mobile health interventions has increased in popularity for the prevention and treatment of eating disorders, a significant amount of research that has been conducted has examined the use of mobile applications (88). Further, to date, there has been limited testing of the use of reciprocal text messaging in a GSH intervention. The results of the present study suggest that continued efforts to examine text messaging in this context are warranted, especially given the desirability of text messaging for many over telephone conversation in this current time period. The use of text messaging may be even more appropriate for underserved populations (e.g., low-income groups) than mobile applications, given that it typically does not require access to the Internet and thus overcomes certain accessibility concerns (89).

#### Theoretical and Clinical Implications

Beyond research, the results of the present study also hold implications for future clinical theory and application. In particular, this investigation lends partial support to the AMWESR model's (50–53) assertion that a focus on mindful self-care will not only support adaptive functioning and positive body image, but also may alleviate disordered eating and related processes. The primary focus of the present intervention was on the internal self-system in this model (i.e., thoughts and feelings about the body). By increasing their active, intentional participation in mindful self-care, participants were able to engage in a type of behavior which their body image concerns would have limited in the past, thus interrupting a vicious cycle in which the body is seen as somehow unworthy of this type of kindness. Future work may wish to continue exploration of how mindful self-care impacts not only the internal system, but the alignment between internal and external aspects of the self as conceptualized in the AMWESR model (e.g., navigating discrepancy between cultural weight stigma and own body image).

Beyond provision of support for existing theory, the success of providing treatment in the GSH format is particularly intriguing given that it lends itself to greater treatment accessibility for groups which have been underserved in the past. The intervention could be made even more scalable and sustainable through use of an automated support person, although it is unclear how this would impact participant response to the program (especially as participants responded favorably to the supporter element). This study also directly relates to broader implications for the use of bibliotherapy and self-help books with clients and patients. Although bibliotherapy is a common recommendation by providers across the healthcare system, at times exploration of the utility and efficacy of these books (especially when recommended in an unguided, unstructured manner) has been lacking (90). Beyond the efficacy of self-help approaches, it is also vital that these programs are tested to determine whether these approaches are contraindicated for patient use. This could occur through direct worsening of symptomatology or waste of patient time and resources which could be dedicated to accessing proven intervention methods. Moreover, relevant to accessibility, there has been a shift to increased use of telehealth during the COVID-19 pandemic. The present study provides preliminary support for a program which can be safely administered in a remote manner.

## Conclusion

Innovative strategies for addressing risk factors for disordered eating are an important area of focus. The present study provides support for the feasibility and acceptability of a novel, text message-facilitated GSH approach in addressing body image concerns of emerging adult women. This approach not only sought to impact risk factors, but also employed an inclusive strategy to also support adaptive functioning through the encouragement of mindful self-care. Although this study is not without limitations, it does provide initial groundwork for future, more rigorous testing of this approach. Ideally, this will broaden prevention efforts and allow for groups which have been limited in the past by issues of accessibility to benefit from these approaches.

## Data Availability Statement

The raw data supporting the conclusions of this article will be made available by the authors, without undue reservation.

## Ethics Statement

The studies involving human participants were reviewed and approved by University of North Carolina at Charlotte Institutional Review Board. The patients/participants provided their written informed consent to participate in this study.

## Author Contributions

All authors listed have made a substantial, direct, and intellectual contribution to the work and approved it for publication.

## Funding

This content first appeared as part of the CR's dissertation study (91) and was funded by the University of North Carolina at Charlotte Thomas L. Reynolds Graduate Student Research Award. Publication fees were covered by the University of North Carolina at Charlotte. The project would not have been possible without consultation with committee members Erika Montanaro, Maggie Quinlan, and Susan Johnson. Erin Thomas also provided guidance regarding the qualitative portion of the study. Research assistants Gretel Maya Farfan and Emorie Worthington provided excellent administrative support.

## Conflict of Interest

The authors declare that the research was conducted in the absence of any commercial or financial relationships that could be construed as a potential conflict of interest.

## Publisher's Note

All claims expressed in this article are solely those of the authors and do not necessarily represent those of their affiliated organizations, or those of the publisher, the editors and the reviewers. Any product that may be evaluated in this article, or claim that may be made by its manufacturer, is not guaranteed or endorsed by the publisher.
